# Health service utilisation during the last year of life: a prospective, longitudinal study of the pathways of patients with chronic kidney disease stages 3-5

**DOI:** 10.1186/s12904-018-0310-8

**Published:** 2018-04-05

**Authors:** Shirley Chambers, Helen Healy, Wendy E. Hoy, Adrian Kark, Sharad Ratanjee, Geoffrey Mitchell, Carol Douglas, Patsy Yates, Ann Bonner

**Affiliations:** 1 Faculty of Health, Queensland University of Technology, Brisbane, Australia; 2 National Health and Medical Research Council, Centre for Research Excellence in End of Life Care, Brisbane, Australia; 3 Kidney Health Service, Metro North Hospital and Health Service, Queensland Health, Brisbane, Australia; 4 National Health and Medical Research Council, Chronic Kidney Disease Centre for Research Excellence, Brisbane, Australia; 5 Faculty of Medicine, University of Queensland, Brisbane, Australia; 6 Palliative Care Service, Royal Brisbane and Women’s Hospital, Queensland Health, Brisbane, Australia; 7 Centre for Palliative Care Research and Education, Queensland Health, Brisbane, Australia

**Keywords:** Chronic kidney disease, Palliative care, Supportive care, Conservative care, End of life, Patient tracking, Health service utilisation, Critical events

## Abstract

**Background:**

Chronic kidney disease (CKD) is a growing global problem affecting around 10% of many countries’ populations. Providing appropriate palliative care services (PCS) to those with advanced kidney disease is becoming paramount. Palliative/supportive care alongside usual CKD clinical treatment is gaining acceptance in nephrology services although the collaboration with and use of PCS is not consistent.

**Methods:**

The goal of this study was to track and quantify the health service utilisation of people with CKD stages 3-5 over the last 12 months of life. Patients were recruited from a kidney health service (Queensland, Australia) for this prospective, longitudinal study. Data were collected for 12 months (or until death, whichever was sooner) during 2015-17 from administrative health sources. Emergency department presentations (EDP) and inpatient admissions (IPA) (collectively referred to as critical events) were reviewed by two Nephrologists to gauge if the events were avoidable.

**Results:**

Participants (*n* = 19) with a median age of 78 years (range 42-90), were mostly male (63%), 79% had CKD stage 5, and were heavy users of health services during the study period**.** Fifteen patients (79%) collectively recorded 44 EDP; 61% occurred after-hours, 91% were triaged as imminently and potentially life-threatening and 73% were admitted. Seventy-four IPA were collectively recorded across 16 patients (84%); 14% occurred on weekends or public holidays. Median length of stay was 3 days (range 1-29). The median number of EDP and IPA per patient was 1 and 2 (range 0-12 and 0-20) respectively. The most common trigger to both EDP (30%) and IPA (15%) was respiratory distress. By study end 37% of patients died, 63% were known to PCS and 11% rejected a referral to a PCS. All critical events were deemed unavoidable.

**Conclusions:**

Few patients avoided using acute health care services in a 12 month period, highlighting the high service needs of this cohort throughout the long, slow decline of CKD. Proactive end-of-life care earlier in the disease trajectory through integrating renal and palliative care teams may avoid acute presentations to hospital through better symptom management and planned care pathways.

## Background

The prevalence and burden of chronic kidney disease (CKD) is increasing globally [1], and is a public health problem affecting more than 10% of people in advanced economies [2]. CKD is characterised by a sustained reduction in kidney function (estimated glomerular filtration rate [eGFR] ≤60 ml/min/1.73m^2^ [3] with the leading causes being diabetes and hypertension [4]. In Australia, where the prevalence of CKD is around 10% [5], the greatest growth in incidence is in those aged over 65 years [6] who tend to have more comorbid conditions, and greater frailty [7], collectively translating to a greater need for health care services [8].

Health care utilisation patterns map a range of activities in the delivery of health care including service planning, resource allocation and analyses/research [9]. National health statistics, such as from admitted patient data [10–12], health expenditure data [13] and other major reports of activities [14], provide a glimpse of how some patient groups use health services. For example, Medicare expenses for CKD in the USA were around $50 billion USD in 2013, accounting for nearly 20% of all Medicare spending [14]. In the UK (2009-10) CKD stages 3-5 alone cost the National Health Service (NHS) around £1.45 billion annually, which equates to around 1.3% of all NHS spending [15]. In Australia CKD is associated with 17% of all hospitalisations [5]. Ngu et al. found that inpatient admissions (IPA) due to CKD in Australia significantly increased over a four-year period from 5.2% to 8.6% of all hospital admissions, and of intensive care admissions from 8.3% to 13.3% [11]. CKD is often related to, or is an underlying condition in, many other admitted disease groups, such as cardiovascular and diabetes [14]. It is a signal for heavy use of health services, reflecting the medical complexity of this group of patients [8].

There is compelling evidence that early integration of palliative care into usual cancer care provides benefits for patients and health systems [16]. While palliative care services (PCS) support patients in their terminal phases of cancer, the seamless integration of PCS into other clinical teams is less established [17]. It has been estimated that, globally, adults with non-malignant conditions make up around 65% of those needing palliative care [18] even though patients with a cancer diagnosis still dominate PCS utilisation [19]. The demand from people with terminal failure across a range of solid organs is also growing in Australia although access to PCS is low with only about 30% of the total PCS being provided to non-cancer patients [20]. In response palliative care is now broadening its scope to include these patient groups [21], notwithstanding the paucity of data about their specific palliative care needs [22]. Palliative care is an important care pathway in advanced CKD (eGFR < 30 ml/min/m^2^) as well as end stage kidney disease (ESKD eGFR < 15 ml/min/m^2^) to manage high symptom burden [23] and the comorbid disease loads in a population characterised by increasing age and frailty. Moreover, ESKD differs from most other terminal organ failure, where, for patients across the lifespan, dialysis can artificially prolong life for decades unlike other devices [24]. For instance, ventricular assist devices are restricted to a far narrower range of patients and are less readily available in many countries. Alternatively, conservative care is another management pathway where patients decide to not commence, or have been medically advised against, dialysis. Patients may also move from the dialysis to the conservative care pathway to stop dialysis [25]. In patients who opt not to have dialysis and instead follow a conservative care pathway, 20% are still alive 3 years later [26]. Compared with terminal cancer, the patterns of health service use [21, 27] and functional decline [28] in advanced CKD are less predictable. These are all important points of differentiation likely to necessitate the re-design of cancer focused PCS to align with the needs of the CKD population. An approach that is gaining acceptance in nephrology teams is combining palliative care and nephrology expertise to create transdisciplinary skillsets particularly important in the domains of symptom control and medication management [24]. The patients’ use and experiences of health services are likely key outcomes of this re-design. This study aimed to understand the health service utilisation patterns in a cohort with advanced CKD whose prognosis was less than a year of life. The results will be translated into the redesign of appropriate palliative care/kidney transdisciplinary teams.

### Aims

This study aimed to quantify patients’ health service use over the 12 month period prior to their (anticipated) death. Specifically, this study reports on emergency department presentations (EDP) and inpatient admissions (IPA) (collectively referred to as critical events) and PCS utilisation. A secondary aim was to report on patients’ symptoms, physical functioning and quality of life over the same period and this has been previously published [29]. Due to the substantial symptom burden [29] and slow functional decline of patients with advanced and progressive CKD, programs that integrate palliative/supportive care into usual clinical kidney care could assist with easing symptom burden particularly in these patients’ last 12 months of life.

## Methods

### Design

For this prospective, longitudinal, observational study, demographic, clinical and health administrative data were collected at regular intervals from patients’ medical records and from hospital-based administrative health databases for a 12 month period, or until their death.

### Setting and sample

Nephrologists (HH, SR and AK) identified potential patients for this study from a kidney health service within the Metro North Hospital and Health Service (Queensland, Australia) throughout 2015-16. The hospitals frequented by the patients are all situated within the same Queensland Health Hospital and Health Service, and are referred to as Hospitals 1 (which has a consultancy only PCS on site), 2 and 3 (which both have a PCS and a dedicated palliative care inpatient unit on site). Hospital 1 is a major referral tertiary hospital (1000-bed) for Hospitals 2 and 3. This hospital provides comprehensive renal care. Hospital 2 is a 250-bed regional hospital that has an onsite satellite dialysis unit and outpatient services but not the other components of a renal service. Hospital 3 is a major teaching hospital (624-bed) that does not have a dedicated renal unit. Hospitals 2 and 3 do not have the capacity to deal with complex renal emergencies so any presenting patients are transferred to the renal service at Hospital 1.

The convenience sample were eligible if they were English speaking adults (≥18 year of age), with CKD stages 3-5 with a prognosis of < 12 months (clinician identified by answering ‘no’ to the surprise question, “would you be surprised if the patient died in the following 12 months?”), and were cognitively sound and competent to give informed consent. Exclusion criteria were extreme psychological or social distress likely to bias the collection of interviewer administered surveys (determined by clinical and/or research staff), patients who died within 48 h of qualifying for the study, or who resided > 2 h’ drive from the recruitment site (to enable in-person data collection). Patients included those who were receiving conservative care (CKD stages 4 and 5) and those receiving dialysis. Our target was to track 20 patients.

### Data sources and tools

Clinical and administrative health data were collected from Queensland Health sources at the appropriate intervals throughout the study period to capture patients’ medical history, emergency department presentations (EDP), inpatient admissions (IPA), and referrals to other health services. Comorbidities were collected from patients’ medical records. See Tables 1 and 2 for details.

**Table 1:** Demographic, clinical and administrative health data collected and timing

Data Collected	Data Collection Time Points		
	Study Entry	3Mthly^a^	Study End
Demographic details	√		√
Medical history	√	√	√
Emergency department presentations		√	√
Inpatient admissions		√	√
Other health service use		√	√
Co-morbidities			√

^a^Cycle continues until patient’s death

**Table 2:** Clinical and administrative health data sources (Queensland Health) and corresponding information retrieved

Data Source *(Administering department)*	Information Collected
Emergency Department Information System (EDIS)	*Emergency department presentations:* presentation date and timing, hospital location, demographic details, mode of arrival (e.g., ambulance), triage category^a^, reason for presentation and departure status
Hospital Based Corporate Information System (HBCIS)	*Inpatient admissions*: admission date, hospital location, demographic details, care type, admission status, reason for admission (AR-DRG), length of stay, discharge date and destination
Medical records^b^	Health history (including comorbidities), diagnostic and treatment details, demographic details, health service referrals and use, and other relevant details

*AR-DRG* Australian Refined-Diagnostic Related Group [30]

^a^Triage categories: 1: immediately life-threatening; 2: imminently life-threatening; 3: potentially life-threatening; 4: potentially serious; 5: less urgent [31]

^b^Hard copy or electronic

### Expert consultant review of critical events

Critical events for the purpose of this study refer to: a) emergency department presentations (EDP); and b) inpatient admissions (IPA). Two nephrologists (AK and SR) reviewed patients’ critical events retrospectively to determine if the events were avoidable or not. They were provided with a summary of each critical event alongside comprehensive chart notes of medical (diagnostic and treatment), social and psychological details, and additional health service utilisation around each event. This was a subjective process using the experience and expertise of the reviewing medical practitioners.

### Analysis

Unique study codes were assigned to the patients on study entry. Descriptive statistics and frequency distributions were generated from the patients’ demographic and clinical characteristics and service usage. Data sourced from Queensland Health administrative databases were triangulated with medical chart notes to identify and resolve discrepancies. Triggers to EDP were identified by collating and categorising the ‘Reason for Presentation’ and other clinical notes as found in EDIS. Triggers to IPA were identified by collating and categorising the Australian Refined-Diagnostic Related Groups (AR-DRG) [30], as recorded in HBCIS, and associated medical notes extracted from patient charts of each admission. Authors AB (expert renal nurse) and SC (non-clinical researcher) collaborated to ensure clinical information was categorised appropriately. Comorbidity scores were calculated using the Charlson Comorbidity index [32].

## Results

Of the 49 potentially eligible patients identified we recruited our target of 20 with two withdrawing within the first 2 months. They were replaced with two more patients from the potentially eligible group. One further patient withdrew at month 4, leaving a final sample of *n* = 19.

### Demographic characteristics

Patients’ median age at study entry was 78 years (range 42-90). Most patients were male (63%), married/defacto (53%) and co-residing with others (84%). Most were in CKD stage 5 (79%). Seven patients (37%) died during the study; of these, one died from a non-CKD related event (car accident). Nine patients (47%) were receiving conservative care throughout the study and the remaining 10 were on a haemodialysis pathway (53%). See Table 3 for full details.

**Table 3:** Patients’ demographic, diagnostic, comorbidity and death details

Characteristics	N = 19 All *N (% All)*	*n* = 9 Conservative *n (% Group)*	*n* = 10 Dialysis *n (% Group)*
Gender			
Male	12 (63)	5 (56)	7 (70)
Age (years)			
Median (range)	78 (42-90)	86 (72-90)	74 (42-90)
< 60 years	3 (16)	0 (0)	3 (30)
60-80	8 (42)	3 (33)	5 (50)
> 80	8 (42)	6 (67)	2 (20)
Marital status			
Married/Defacto	10 (53)	5 (56)	5 (50)
Living arrangements			
Lives with others	16 (84)^a^	7 (78)^b^	9 (90)^c^
Lives alone	3 (16)	2 (22)	1 (10)
CKD stage			
Stage 4	4 (21)	4 (44)	–
Stage 5	15 (79)	5 (56)	10 (100)
Comorbidity index			
Median (range)	8 (3-11)	9 (7-10)	6.5 (3-11)
Deaths during study period			
Number^d^	7 (37)	4 (44)	3 (30)
Median (range) days from study entry^d^	148 (92-330)	217 (92-330)	103 (101-228)

*Note:* Percentages may not equal 100% due to rounding,

^a^Three patients lived in nursing homes and one patient shared a house - not with a carer

^b^Two patients lived in nursing homes

^c^One patient lived in a nursing home and one patient shared a house - not with a carer

^d^Includes a patient who died as a result of an accident

### Emergency department presentations (EDP)

Fifteen patients recorded 44 emergency department presentations (EDP) collectively at the participating hospitals. Patients receiving conservative care were more likely to present to Hospital 2 whereas those receiving haemodialysis presented to Hospital 1. Place of residence can dictate which hospital the patient is taken to in an emergency, which may explain the four hospital transfers where patients presented to one emergency department (ED) and then were transferred onto Hospital 1.

Patients in the dialysis group presented to the ED after-hours more often than those in the conservative group (43% versus 16% respectively). Most patients (89% of EDP) arrived at the ED by ambulance. These EDP were significant with 40 EDP (91%) triaged as categories 2 and 3 (Imminently and potentially life-threatening respectively) [32] and 73% admitted, i.e., transition to IPA. Patients spent considerable time in the ED (median 6.4 h [range 1.8 to 24.8 h]). Seven patients (42%) presented at EDs three or more times during the study period. Frequent attendance at ED was less common in the conservative group (*n* = 2/9; 22% of group) compared to the dialysis group (*n* = 5/10; 50% of group). See Table 4 for additional detail.

**Table 4:** Emergency department Presentations (EDP) during study period^a^

Variables of Interest	*N* = 19 All 44 EDP *# EDP (% All EDP)*	*n* = 9 Conservative 13 EDP *# EDP (% Group’s EDP)*	*n* = 10 Dialysis 31 EDP *# EDP (% Group’ EDP)*
Hospital			
1	26 (59)	2 (15)	24 (77)
2	17 (39)	11 (85)	6 (19)
3	1 (2)	0 (0)	1 (3)
Main modes of arrival			
Ambulance	39 (89)	10 (77)	29 (94)
Walk in	4 (9)	3 (23)	1 (3)
After-hours presentations^b^	26 (61)	7 (16)	19 (43)
Triage category			
Category 1	0 (0)	0 (0)	0 (0)
Category 2	17 (39)	1 (8)	16 (52)
Category 3	23 (52)	10 (77)	13 (42)
Category 4	3 (7)	2 (15)	1 (3)
Category 5	1 (2)	0 (0)	1 (3)
Departure status			
Admitted	32 (73)	9 (69)	23 (74)
Transfer to other hospital	4 (9)	1 (8)	3 (10)
Home	4^c^ (9)	3 (23)	1^2^ (3)
Dialysis clinic	4 (9)	N/A	4 (13)
Length of stay in ED (hours)			
Median (range)	6.4 (1.8 - 24.8)	5 (3.1 - 13.1)	1.5 (1.8 - 24.8)
Number of EDP per patient			
Median (range)	1 (0-12)	1 (0-4)	2 (0-12)
	*n (% All Patients)*	*n (% Group)*	*n (% Group)*
0	4 (21)	2 (22)	2 (20)
1-2	8 (42)	5 (56)	3 (30)
3-5	6 (32)	2 (22)	4 (40)
6 +	1 (5)	0 (0)	1 (10)

*Note:* Totals may not add up to 100% due to rounding

*EDP* Emergency department presentation/s

^a^As per EDIS data

^b^After-hours defined as between the hours of 5 pm and 7 am on week days plus all weekend days and public holidays

^c^Includes one patient who declined admission

#### Triggers to EDP

The most common triggers for EDP were respiratory distress, pain (which was separated into chest pain and other pain), hypotension and falls. Respiratory distress was the most common trigger for EDP for the dialysis group (32% of EDP) whereas pain was the most common trigger in the conservative group (38% of EDP). See Table 5 for more details.

**Table 5:** Five most often recorded triggers to emergency department presentations (EDP)

Triggers^a^	*N* = 19 All *# EDP (% All EDP)*	n = 9 Conservative *# EDP (% Group’s EDP)*	*n* = 10 Dialysis *# EDP (% Group’s EDP)*
Respiratory distress	13 (30)	3 (23)	10 (32)
Pain (other than chest)	8 (18)	5 (38)	3 (10)
Chest pain	6 (14)	1 (8)	5 (16)
Hypotension	6 (14)	1 (8)	5 (16)
Falls	5 (11)	4 (31)	0 (0)

*EDP* Emergency Department Presentation/s

^a^These triggers are not mutually exclusive, i.e., the patient may have presented with more than one trigger

### Inpatient admissions (IPA)

During the study period, 16 patients (84%) recorded 74 inpatient admissions (IPA) collectively, excluding routine dialysis admissions, across participating hospitals. Patients receiving dialysis were principally admitted to Hospital 1 (90% of group’s IPA). The conservative group recorded a similar number of IPA on weekends or public holidays (13%), as the dialysis group (14%). Most of the IPA were sourced via an ED (49%) although some patients were sent to an outpatient renal clinic or the dialysis unit from an ED so the source of their admissions was classified in hospital databases from those areas. There were other patients who had routine dialysis and then were admitted to an acute ward. Most admissions (58%) were classified as an ‘emergency’. The code descriptor *Episode Change* (also known as a statistical admission) can be used to document a change in the patient’s Care Type [30], for example from acute to palliative, rehabilitation or maintenance, and back to acute, while not being physically moved from the hospital ward, and to describe the IPA source, admission status or discharge destination. In this study episode changes were the source of few IPA (8%). Some patient transfers were from hospitals not participating in the study hence the corresponding EDP were not captured. Most Care Types were recorded as *Acute* (92% of IPA), with very few recorded as *Palliative* (5% IPA).

Most IPA resulted in patients returning home (76%). Five of the six illness-related deaths of study patients were recorded at the participating hospitals. If a patient did not die in a recruitment hospital, and we were not informed of their place of death by relatives (carers), their place of death for the purpose of this study is unknown. The length of stay of IPA was similar for all patients combined (Median 3 days; range 1-29) and per group. Of particular interest, *n* = 14 patients recorded multiple IPA during the study period. See Table 6 for additional information relating to the patients’ IPA during the study period.

**Table 6:** Inpatient admissions (IPA) during study period^a^

Variables of Interest	N = 19 All 74 IPA^b^ *# IPA (% All IPA)*	n = 9 Conservative 16 IPA *# IPA (% Group’s IPA)*	n = 10 Dialysis 58 IPA^b^ *# IPA (% Group’s IPA)*
Hospital			
1	59 (80)	7 (44)	52 (90)
2	14 (19)	9 (56)	5 (9)
3	1 (1)	0 (0)	1 (2)
Weekend/public holidays	10 (14)	2 (13)	8 (14)
Source of IPA			
Emergency Department	36 (49)	10 (63)	26 (45)
Outpatient Department	15 (20)	4 (25)	11 (19)
Transfer	9 (12)	1 (6)	7 (12)
Routine IPA	8 (11)	0 (0)	9 (16)
Episode change	6 (8)	1 (6)	5 (9)
Admission status			
Emergency	43 (58)	10 (63)	33 (57)
Elective	15 (20)	5 (31)	10 (17)
Not assigned	15 (20)	1 (6)	14 (24)
Episode change	1 (1)	0 (0)	1 (2)
Care type			
Acute	68 (92)	15 (94)	53 (91)
Palliative	4 (5)	1 (6)	3 (5)
Maintenance	1 (1)	0 (0)	1 (2)
Rehabilitation	1 (1)	0 (0)	1 (2)
Discharge destination			
Home	56 (76)	11 (69)	45 (78)
T ransfer	6 (8)	1 (6)	5 (9)
Episode change	6 (8)	1 (6)	5 (9)
Died	5 (7)	2 (13)	3 (5)
Aged care facility (initial)	1 (1)	1 (6)	0 (0)
Length of stay (days)			
Median (range)	3 (1-29)	3.5 (1-29)	3 (1-26)
Number of IPA per patient			
Median (range)	2 (0-20)	2 (0-3)	5 (0-20)
	N (% *All Patients)*	*n (% Group)*	*n (% Group)*
0	3 (16)	1 (11)	2 (20)
1-2	8 (42)	6 (67)	2 (20)
3-5	3 (16)	2 (22)	1 (10)
6+	5 (26)	0 (0)	5 (50)

*Note:* Totals may not add up to 100% due to rounding

*IPA* Inpatient admission/s

^a^As per HBCIS data [30]

^b^Excluding routine dialysis admissions

#### Triggers to IPA

The most often recorded triggers for IPA were similar to those for EDP; this is not surprising as most EDP (73%) resulted in an IPA. These triggers were respiratory distress, chest pain, cardiac events, ‘other’ pain, and vascular access issues such as fistula repairs. See Table 7 for more details.

**Table 7:** Five most often recorded triggers for inpatient admissions (IPA)

Trigger^a^	All N = 19 *# IPA (% All IPA)*	Conservative n = 9 *# IPA (% Group’s IPA)*	Dialysis n = 10 *# IPA (% Group’s IPA)*
Respiratory distress	11 (15)	0 (0)	11 (19)
Chest pain	8 (11)	1 (25)	7 (14)
Cardiac event	6 (8)	0 (0)	6 (10)
Other pain	6 (8)	2 (13)	4 (7)
Vascular access issues	6 (8)	0 (0)	6 (10)

*IPA* Inpatient Admission/s

^a^These triggers are not mutually exclusive, i.e., the patient may present with more than one trigger

### Consultant review of critical events

All critical events were deemed to be unavoidable taking into consideration the context of each patient’s clinical presentation, treatment care pathway and social situation.

### Palliative care service referral status

During this study, the kidney health service which spans the three study sites received funding to commence an integrated Kidney Supportive Care program (KSCp) (comprised of a renal and palliative care multidisciplinary team) which is provided earlier in the CKD trajectory to primarily focus on symptom management and decision-making. This program interfaces with the renal treating team, the patient’s general practitioner, and when required, refers seamlessly to PCS. Table 8 provides details of referrals to PCS or to the new KSCp. Two patients undergoing conservative treatment were engaged with a PCS at study entry. Six of the seven patients who died during their study participation were referred to a PCS either prior to or during the study. Only one patient was referred to both services. Twelve patients were referred to and/or were receiving care from either of these services by the time of their death or by study end. Two patients (both receiving dialysis), who were still living at study end, were offered a referral to the KSCp but rejected the suggestion reporting they were not ready for supportive care discussions. Referrals to PCS were received very close to three patients’ deaths (3, 11 and 16 days prior to death).

**Table 8:** Palliative care referral status of patients across study period

Palliative care status	All N = 19 *n (% All Patients)*	Conservative *n* = 9 *n (% Group)*	Dialysis n = 10 *n (% Group)*
Known to PCS at study entry	4^a^ (21)	4^a^ (44)	0 (0)
Referred to KSCp during study	5^b^ (26)	1^b^ (11)	4 (40)
Referred to PCS during study	5^c^ (26)	3^c^ (33)	2 (20)
Total known to PCS/KSCp by study end	12 (63)	6 (67)	6 (60)
Refused referral to KSCp	2 (11)	0 (0)	2 (20)

*PCS* Palliative Care Service, *KSCp* Kidney Supportive Care Program

^a^Two of these patients were not actively engaged with a PCS at study entry

^b^One of these patients was already known to a PCS at study entry

^c^Two of these patients were known to, but were not actively engaged with, a PCS at study entry

## Discussion

We prospectively followed each patient, anticipated to be in their last year of life, for a maximum of 12 months to describe critical health service events and the use of PCS, and while the sample was small, it does reflect the heterogeneity of the advanced CKD population at this site. The study cohort were heavy users of health services as a whole, however the dialysis subgroup in particular were responsible for most of the health service use during the study period. Almost all study patients had at least one EDP with most arriving at the ED by ambulance followed by admission to an acute ward. All critical events were unavoidable, and many critical events occurred after-hours. The most frequent trigger for EDP and IPA was respiratory distress. Most patients who died during the study were engaged with a PCS prior to death although overall there were fewer deaths than anticipated.

Previous studies identifying patterns in health service utilisation in those with CKD are limited by their retrospective design, reliance on various forms of administrative health and insurance data [8, 11, 33] and/or incomplete capture of individual patient-level data. These limitations are shared by previous Australian studies of service use near the end of life [34–37]. Unlike reports from the Australian Hospital Statistics [10, 12, 38] which report collated service utilisation data (with the unit of measure being the occasion of service), this study reports the type as well as the number of critical events experienced by individual patients during their study participation.

Several other studies report people with advanced CKD are high users of health services [8, 11, 39]. The later stages of CKD is characterised by multiple comorbidities, [40], high symptom burden [23, 41–44] and a highly variable premature life expectancy [45–48]. Our findings of multiple critical events recorded for individual patients during the study period align with the literature. Patients receiving dialysis recorded more critical events per patient than the conservative group, excluding routine dialysis admissions, even though they were younger than the conservative group. Typically the group of patients who opt for conservative care tend to be on average older with more comorbidities [26, 49] than those who opt for dialysis. While Quinn et al. found fewer IPA per patient in a haemodialysis cohort than our study, they did find that patient groups who are known to be heavy users of inpatient services, such as cardiology, had fewer IPA than patients with ESKD [39]. However Quinn and colleagues did not select patients on the basis of a prognosis of less than 12 months [39]. Nevertheless, both our study and Quinn’s report relatively high consumption of health resources by patients with advanced CKD. Our study is not representative of the Australian population which showed EDP by ambulance was lower at 24% in 2015-16 Australia wide compared to 89% in our study [50]. Furthermore, around 29% of all EDP in Australia during this time resulted in an IPA [50] compared with 73% of our cohort, highlighting the high service needs of the advanced CKD cohort.

The duration of IPA in CKD cohorts varies widely across studies [21, 39, 51]. These studies are heterogeneous, with differences including study patient selection. We did, however, find that the median length of stay of IPA in our study group was shorter than the Australian average of 5.5 days [12] notwithstanding the study selection criterion of the last 12 months of life. These differences in duration of IPA may reflect whole of hospital systems and practices at the study sites. Of interest, in those who died during the study, four had a palliative care related IPA. Given that all patients were expected to die within 12 months and had substantial symptom burden [29], these findings confirm the literature in that this patient group are not being referred early enough for palliative care/community services [52] even though in Australia, withdrawal from haemodialysis is the leading cause of death in these patients [53].

The after-hours initiated critical events captured during the study period are high. This may be because patients lack access to and/or knowledge of appropriate support, such as 24 h community services (e.g., general practitioners and domiciliary nursing services) and advice. In the area where this study took place, 24 h palliative care nursing care is available, however it is limited to those who are in the terminal phase of their disease. Another explanation is the complexity of the study patient cohort, with all critical events recorded during this study deemed to be unavoidable. We found that respiratory distress was the most common trigger to both EDP and IPA compared to findings in other studies of patients with CKD [8] or cancer [54] where pain was more likely to trigger an EDP. Patients with advanced CKD will benefit from palliative care input to initiate end-of-life discussions and to help determine future goals of care [55] such as their preferred place of care and withdrawal from dialysis. Furthermore, in collaboration with renal services, PCS can provide support with symptom management, and psychological support and education for both the patient and carer to manage expected exacerbation of symptoms such as breathlessness and pain [24]. Access to PCS earlier in the illness trajectory, rather than in the terminal phase, will ensure end-of-life decisions are made without urgency [56]. Proactive planning by PCS to address known triggers that could lead to acute health service use, particularly for events that occur after hours, are likely to reduce patients’ use of these services or at the least reduce the length of IPA [57].

There are limitations inherent when using administrative health data to inform research as these data are primarily gathered for financial, administrative management and departmental reporting purposes [58]. We found that patient transitions through acute services were often not transparent. For example, the percentage of EDP that resulted in an IPA did not equate to the percentage of IPA sourced from an ED which is probably due to sourcing two different databases (EDIS and HBCIS) and how the processes of transfers between hospitals, and episode changes, are recorded. We were able to quality assure dataset integrity in our study by validation against chart reviews. However this was only possible because of our small sample size. For example, one patient who presented to the ED of Hospital 2 was admitted to the ED Short Stay Unit while waiting for a transfer to Hospital 1 for specialist renal care. Hence, the admission source of the IPA at Hospital 1 was classified in the inpatient database (HBCIS) as a ‘Transfer’ rather than an admission from the ‘Emergency Department’. Furthermore, the Admission Status code of *Not Assigned* does not provide any information of the nature of the IPA, such as emergency or elective. Research using large datasets is critically dependent on data integrity and the methodological approach used may lead to underestimating ED use and other health service use in the CKD patient population.

Major strengths of this study include its prospective, longitudinal design and our recruitment method. We recruited patients directly from a kidney service. Hence the patients were already known to have underlying CKD on study entry. Relying on administrative health data alone to identify cases by International Classification of Diseases (ICD-10) codes [59] or *Palliative Care* occasions of service (by classifications and categories) includes patients who develop renal failure secondary to other health conditions rather than having a pre-existing CKD diagnosis. Furthermore, we report on patients’ multiple critical events during the study period and capture the triggers to, and geographical location of, these events. Nevertheless, there are also limitations to our study. Due to the small convenience sample, findings cannot be generalised. Bias may have been introduced into the study as: i) the patients were recruited from one kidney service, ii) recruitment was limited to patients who resided < 2 h drive from the recruitment site, iii) three investigators (HH, AK, and SR) were treating clinicians, and iv) two of these nephrologists (AK and SR) also reviewed the critical events. This has the potential to be both a limitation and strength of the study due to the nephrologists’ extensive knowledge of the treatment used at the recruitment sites and/or of patients’ specific conditions. Even though the patients in this study were chosen due to their limited prognosis, less died than would have been anticipated with a 12-month prognosis. A recent study found prognostication using the ‘surprise question’ in those in stages 4-5 CKD had moderate sensitivity and specificity (55% and 76% respectively) [60] which may explain why fewer participants died during our study**.** Therefore, comparison to previous studies (of patients nearing the end of life) should be treated with caution as previous studies may have selected their study population differently, for example, persons in the last 3 or 6 months of life. Despite these limitations, the findings provide insight into the critical events contributing to health service use. Undertaking this study proved the feasibility of i) recruiting a vulnerable CKD cohort, and ii) collecting health administrative data prospectively, that is, in almost ‘real time’ from a variety of health administrative sources. Furthermore, the triangulation of these data with comprehensive chart notes provided precision at the level of data elements of patients’ complex health service utilisation.

## Conclusion

The purpose of this study was to follow and quantify the use of health services by patients with CKD in their last 12 months of life. Most study patients were heavy users of acute health services which highlights their complex needs. Usage is validated by the distressing and potentially life threatening character of the triggers to the critical events. Therefore this cohort needs matching high quality care to manage their complex conditions and to avoid or reduce after-hours critical events. Nephrology experts worldwide note that the quality of care, particularly conservative and palliative care, is currently suboptimal for persons with advanced CKD [56]. It is therefore imperative to assemble timely, effective and sustainable high quality care pathways that meet patient need. Our findings will inform the design of, and methods used in, future multisite studies to provide evidence to inform the development of timely palliative care models that improve patient outcomes for those with advanced stages of CKD.

### Implication for practice

The CKD population is heterogeneous in end-of-life trajectories with those in the conservative care group typically experiencing a long and slow decline of many months to several years to death whereas those who withdraw from haemodialysis die within 7-10 days. Traditional siloed models of renal and palliative care teams are the dominant model; re-design of clinical services is required to better match the needs of these vulnerable patients. For instance in the UK, one of the leaders in delivering kidney supportive care, only about 23% of renal units have an integrated renal/palliative care team [61], although most restrict this service to the conservative care group of patients. Renal nurses are also strategically placed to assess for symptom burden, increasing frailty, and to coordinate the care of at-risk patients receiving kidney replacement therapies. Referral of patients to integrated renal/palliative care teams earlier in the CKD trajectory may reduce triggers to critical events which contribute to EDP and IPA as well as providing a seamless conduit to specialist PCS when the terminal phase approaches.

## Abbreviations

AR-DRG: Australian Refined-Diagnostic Related Group
CKD: Chronic Kidney Disease
ED: Emergency Department
EDIS: Emergency Department Information System
EDP: Emergency Department Presentation/s
eGFR: Estimated Glomerular Filtration Rate
ESKD: End Stage Kidney Disease
HBCIS: Hospital Based Corporate Information System
ICD-10 codes: International Classification of Diseases-10th Revision
IPA: Inpatient Admission/s
KSCp: Kidney Supportive Care Program
NHS: National Health Service
PCS: Palliative Care Service/s

## References

[1] GBD 2015 Disease and Injury Incidence and Prevalence Collaborators (2016). Global, regional, and national incidence, prevalence, and years lived with disability for 310 diseases and injuries, 1990-2015: a systematic analysis for the global burden of disease study 2015. Lancet.

[2] Eckardt K-U, Coresh J, Devuyst O, Johnson RJ, Köttgen A, Levey AS (2013). Evolving importance of kidney disease: from subspecialty to global health burden. Lancet.

[3] Kidney Disease Improving Global Outcomes (2013). Definition and classification of CKD. Kidney Int Suppl.

[4] Webster AC, Nagler EV, Morton RL, Masson P (2017). Chronic kidney disease. Lancet.

[5] Australian Institute of Health and Welfare. Chronic kidney disease compendium. Web Report 2016. https://www.aihw.gov.au/reports/chronic-kidney-disease/chronic-kidney-disease-compendium/contents/how-many-australians-have-chronic-kidney-disease. Accessed 20 Oct 2016.

[6] Australian Institute of Health and Welfare (2016). Incidence of end-stage kidney disease in Australia 1997-2013.

[7] Kittiskulnam P, Sheshadri A, Johansen KL (2016). Consequences of CKD on functioning. Semin Nephrol.

[8] Welch JL, Meek J, Bartlett Ellis RJ, Ambuehl R, Decker BS (2017). Patterns of healthcare encounters experienced by patients with chronic kidney disease. J Renal Care.

[9] Katz L, Fink RV, Bozeman SR, McNeil BJ (2014). Using health care utilization and publication patterns to characterize the research portfolio and to plan future reserach investments. PLoS One.

[10] Australian Institute of Health and Welfare (2016). Admitted patient care 2014-15: Australian hospital statistics.

[11] Ngu K, Reid D, Tobin A (2017). Trends and outcomes of chronic kidney disease in intensive care: a 5-year study. Intern Med J.

[12] Australian Institute of Health and Welfare (2017). Admitted patient care 2015-16: Australian hospital statistics.

[13] Australian Institute of Health and Welfare (2017). Australian health expenditure - demographics and diseases: hospital admitted patient expenditure 2004-05 to 2012-13.

[14] United States Renal Data System (USRDS). United States renal data system annual data report: epidemiology of kidney disease in the United States. 2015. https://www.usrds.org/2015/view/Default.aspx. Accessed 20 Oct 2017.

[15] Kerr M, Bray B, Medcalf J, O’Donoghue DJ, Matthews B (2012). Estimating the financial cost of chronic kidney disease to the NHS in England. Nephrol Dial Transplant.

[16] Bakitas MA, El-Jawahri A, Farquhar M, Ferrell B, Grudzen C, Higginson I (2017). The TEAM approach to improving oncology outcomes by incorporating palliative care in practice. J Oncol Pract.

[17] Hui D, Elsayem A, De La Cruz M, Berger A, Zhukovsky DS, Palla S (2010). Availability and integration of palliative care at US cancer centres. JAMA.

[18] Connor SR, Bermedo MCSB, Worldwide Palliative Care Alliance (WPCA) (2014). Global atlas of palliative care at the end of life.

[19] National Council for Palliative Care. National survey of patient activity data for specialist palliative care services: minimum data set (MDS) inpatient services trend report for 2014-15. 2015. http://www.ncpc.org.uk/sites/default/files/user/documents/MDS%20Inpatients%20final%20report%202014_2015_1.pdf. Accessed 30 Nov 2017.

[20] Rosenwax L, Spilsbury K, McNamara BA, Semmens JB (2016). A retrospective population based cohort study of access to specialist palliative care in the last year of life: who is still missing out a decade on?. BMC Palliat Care.

[21] Brady B, Redahan L, Donohoe CL, Mellotte GJ, Wall C, Higgins S (2017). Renal patients at end of life: a 5-year retrospective review. Prog Palliat Care.

[22] Murtagh FE, Higginson IJ (2007). Death from renal failure eighty years on: how far have we come?. J Palliat Med.

[23] Murtagh FE, Addington-Hall JM, Edmonds PM, Donohoe P, Carey I, Jenkins K (2007). Symptoms in advanced renal disease: a cross-sectional survey of symptom prevalence in stage 5 chronic kidney disease managed without dialysis. J Palliat Med.

[24] Kane PM, Vinen K, Murtagh FE (2013). Palliative care for advanced renal disease: a summary of the evidence and future direction. Palliat Med.

[25] Murtagh FE, Burns A, Moranne O, Morton RL, Naicker S (2016). Supportive care: comprehensive conservative care in end-stage kidney disease. Clin J Am Soc Nephrol.

[26] Morton RL, Webster AC, McGeechan K (2016). Conservative management and end of life care in an Australian cohort with ESRD. Clin J Am Soc Nephrol.

[27] Addington-Hall J, Fakhoury W, McCarthy M (1998). Specialist palliative care in nonmalignant disease. Palliat Med.

[28] Walker SR, Brar R, Eng F, Komenda P, Rigatto C, Prasad B (2015). Frailty and physical function in chronic kidney disease: the CanFIT study. Can J Kidney Health Dis.

[29] Bonner A, Chambers S, Healy H, Hoy WE, Mitchell G, Kark A, et al. Tracking patients with advanced kidney disease in last 12 months of life. J Renal Care. 2018; 10.1111/jorc.12239. 10.1111/jorc.12239 29493102

[30] Queensland Health (2017). Queensland hospital admitted patient data collection ( QHAPDC) manual 2017-2018.

[31] Queensland Government Queensland Health. Emergency Department Categories. Emergency Departments 2016. http://www.performance.health.qld.gov.au/hospitalperformance/ed-categories.aspx?hospital=1. Accessed 10 May 2017.

[32] Charlson M, Szatrowski TP, Peterson J, Gold J (1994). Validation of a combined comorbidity index. J Clin Epidemiol.

[33] Mix TCH, Peter WL, Ebben J, Xue J, Pereira BJG, Kausz AT (2003). Hospitalization during advancing chronic kidney disease. Am J Kidney Dis.

[34] O'Connell DL, Goldsbury ED, Davidson P, Girgis A, Phillips JL, Piza M (2014). Acute hospital-based services utilisation during the last year of life in New South Wales, Australia: methods for a population-based study. BMJ Open.

[35] Langton JM, Srasuebkul P, Reeve R, Parkinson B, Gu Y, Buckley NA (2015). Resource use, costs and quality of end-of-life care: observations in a cohort of elderly Australian cancer decedents. Implement Sci.

[36] Rosenwax LK, McNamara BA, Murray K, McCabe RJ, Aoun SM, Currow DC (2011). Hospital and emergency department use in the last year of life: a baseline for future modifications to end-of-life care. MJA.

[37] Rosenwax LK, McNamara BA (2006). Who receives specialist palliative care in Western Australia – and who misses out?. Palliat Med.

[38] Australian Institute of Health and Welfare (2017). Emergency department care 2016-17 Australian hospital statistics.

[39] Quinn MP, Cardwell CR, Rainey A, McNamee PT, Kee F, Maxwell AP (2011). Patterns of hospitalisation before and following initiation of haemodialysis: a 5 year single Centre study. Postgrad Med J.

[40] Fraser SD, Roderick PJ, May CR, McIntyre N, McIntyre C, Fluck RJ (2015). The burden of comorbidity in people with chronic kidney disease stage 3: a cohort study. BMC Nephrol.

[41] Murtagh FE, Addington-Hall J, Edmonds P, Donohoe P, Carey I, Jenkins K (2010). Symptoms in the month before death for stage 5 chronic kidney disease patients managed without dialysis. J Pain Symptom Manag.

[42] Noble H, Meyer J, Bridges J, Bridges J, Kelly D, Johnson B (2008). Patient experience of dialysis refusal or withdrawal - a review of the literature. J Renal Care.

[43] Axelsson L, Alvariza A, Lindberg J, Ohlen J, Hakanson C, Reimertz H (2018). Unmet palliative care needs among patients with end-stage kidney disease: a national registry study about the last week of life. J Pain Symptom Manag.

[44] Almutary H, Bonner A, Douglas C (2013). Symptom burden in chronic kidney disease: a review of recent literature. J Renal Care.

[45] O'Hare AM, Song MK, Kurella Tamura M, Moss AH (2017). Research priorities for palliative care for older adults with advanced chronic kidney disease. J Palliat Med.

[46] Davison SN, Moss AH (2016). Supportive care: meeting the needs of patients with advanced chronic kidney disease. Clin J Am Soc Nephrol.

[47] Makar MS, Pun PH (2017). Sudden cardiac death among hemodialysis patients. Am J Kidney Dis.

[48] Ramesh S, Zalucky A, Hemmelgarn BR, Roberts DJ, Ahmed SB, Wilton SB (2016). Incidence of sudden cardiac death in adults with end-stage renal disease: a systematic review and meta-analysis. BMC Nephrol.

[49] Tonkin-Crine S, Okamoto I, Leydon GM, Murtagh FE, Farrington K, Caskey F (2015). Understanding by older patients of dialysis and conservative management for chronic kidney failure. Am J Kidney Dis.

[50] Australian Institute of Health and Welfare (2016). Emergency department care 2015-16: Australian hospital statistics.

[51] Spilsbury K, Rosenwax L, Arendts G, Semmens JB (2017). The impact of community-based palliative care on acute hospital use in the last year of life is modified by time to death, age and underlying cause of death. A population-based retrospective cohort study. PLoS One.

[52] Redahan L, Brady B, Smyth A, Higgins S, Wall C (2013). The use of palliative care services amongst end-stage kidney disease patients in an Irish tertiary referral Centre. Clin Kidney J.

[53] ANZDATA Registry (2017). 39th report, chapter 3: mortality in end stage kidney disease.

[54] van der Meer DM, Weiland TJ, Philip J, Jelinek GA, Boughey M, Knott J (2016). Presentation patterns and outcomes of patients with cancer accessing care in emergency departments in Victoria, Australia. Support Care Cancer.

[55] Bansal AD, Schell JO (2018). A practical guide for the care of patients with end-stage renal disease near the end of life. Semin Dial.

[56] Davison SN, Levin A, Moss AH, Jha V, Brown EA, Brennan F (2015). Executive summary of the KDIGO controversies conference on supportive care in chronic kidney disease: developing a roadmap to improving quality care. Kidney Int.

[57] Smith S, Brick A, O'Hara S, Normand C (2014). Evidence on the cost and cost effectiveness of palliative care: a literature review. Palliat Med.

[58] Hashimoto RE, Brodt ED, Skelly AC, Dettori JR (2014). Administrative database studies: goldmine or goose chase?. Evid Based Spine Care J.

[59] World Health Organisation. ICD-10: international statistical classification of diseases and related health problems. Volume 2 Instruction Manual. 2010. http://apps.who.int/classifications/icd10/browse/Content/statichtml/ICD10Volume2_en_2010.pdf. Accessed 16 Nov 2017.

[60] Javier AD, Figueroa R, Siew ED, Salat H, Morse J, Stewart TG (2017). Reliability and utility of the surprise question in CKD stages 4 to 5. Am J Kidney Dis.

[61] Okamoto I, Tonkin-Crine S, Rayner H, Murtagh FE, Farrington K, Caskey F (2015). Conservative care for ESRD in the United Kingdom: a national survey. Clin J Am Soc Nephrol.

